# Current perspectives on the dynamic culture of mesenchymal stromal/stem cell spheroids

**DOI:** 10.1093/stcltm/szae093

**Published:** 2024-12-31

**Authors:** Yumi Ohori-Morita, Amal Ashry, Kunimichi Niibe, Hiroshi Egusa

**Affiliations:** Division of Molecular and Regenerative Prosthodontics, Tohoku University Graduate School of Dentistry, 4-1 Seiryo-machi, Aoba-ku, Sendai, Miyagi 980-8575, Japan; Division of Molecular and Regenerative Prosthodontics, Tohoku University Graduate School of Dentistry, 4-1 Seiryo-machi, Aoba-ku, Sendai, Miyagi 980-8575, Japan; Division of Molecular and Regenerative Prosthodontics, Tohoku University Graduate School of Dentistry, 4-1 Seiryo-machi, Aoba-ku, Sendai, Miyagi 980-8575, Japan; Division of Molecular and Regenerative Prosthodontics, Tohoku University Graduate School of Dentistry, 4-1 Seiryo-machi, Aoba-ku, Sendai, Miyagi 980-8575, Japan; Center for Advanced Stem Cell and Regenerative Research, Tohoku University Graduate School of Dentistry, 4-1 Seiryo-machi, Aoba-ku, Sendai, Miyagi 980-8575, Japan

**Keywords:** cell aggregation, dynamic culture, mesenchymal stromal/stem cells, static culture, spheroids

## Abstract

Mesenchymal stromal/stem cells (MSCs) are promising candidates for regenerative medicine owing to their self-renewal properties, multilineage differentiation, immunomodulatory effects, and angiogenic potential. MSC spheroids fabricated by 3D culture have recently shown enhanced therapeutic potential. MSC spheroids create a specialized niche with tight cell-cell and cell-extracellular matrix interactions, optimizing their cellular function by mimicking the in vivo environment. Methods for 3D cultivation of MSCs can be classified into 2 main forms: static suspension culture and dynamic suspension culture. Numerous studies have reported the beneficial influence of these methods on MSCs, which is displayed by increased differentiation, angiogenic, immunomodulatory, and anti-apoptotic effects, and stemness of MSC spheroids. Particularly, recent studies highlighted the benefits of dynamic suspension cultures of the MSC spheroids in terms of faster and more compact spheroid formation and the long-term maintenance of stemness properties. However, only a few studies have compared the behavior of MSC spheroids formed using static and dynamic suspension cultures, considering the significant differences between their culture conditions. This review summarizes the differences between static and dynamic suspension culture methods and discusses the biological outcomes of MSC spheroids reported in the literature. In particular, we highlight the advantages of the dynamic suspension culture of MSC spheroids and contemplate its future applications for various diseases.

## Graphical Abstract

**Graphical Abstract:**
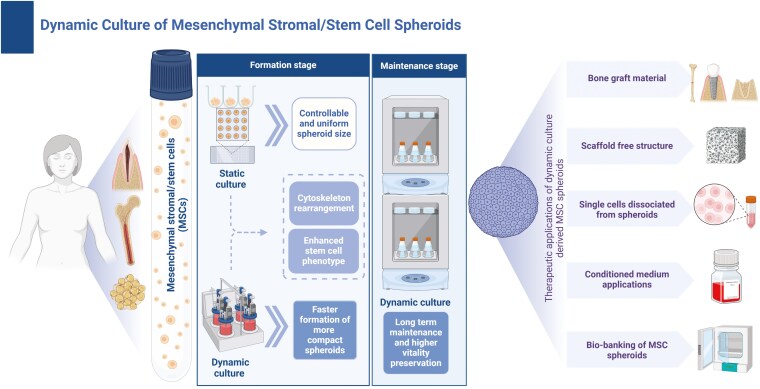

Summarized advantages of dynamic culture mesenchymal stromal/stem cell (MSC) spheroids. Dynamic culture of MSCs enables faster formation of more compact spheroids in the formation stage while achieving long-term maintenance and higher vitality preservation of spheroids in the maintenance stage. These spheroids provide promising tools for the therapeutic use of stem cells for various diseases. Created with BioRender.com/u03l615.

**Significance statement**

Mesenchymal stromal/stem cell (MSC) spheroids have shown enhanced therapeutic potential; however, the difference between MSC spheroids formed using static and dynamic suspension cultures is poorly understood. Recent reports indicate that the benefits of dynamic suspension cultures of the MSC spheroids include faster and more compact spheroid formation and the long-term maintenance of MSC spheroids in terms of their size and stemness properties, which make them attractive for therapeutic applications. This review outlines the current perspectives on the advantages of the dynamic suspension culture of MSC spheroids and illustrates its possible applications for various diseases.

## Introduction

Mesenchymal stromal/stem cells (MSCs) are characterized by their ability to demonstrate proliferative potential when adhering to plastic plates and differentiating into mesenchymal lineages such as osteoblasts, chondrocytes, and adipocytes. However, numerous reports suggest that MSCs cultured using adherent culture methods change stemness during passaging, leading to variability in results between patients and facilities and posing challenges for clinical applications [1]. Biological, chemical, physical, and mechanical cues can profoundly influence the cellular characteristics of MSCs. However, commonly used MSC culture conditions, such as 2D cultivation on plastic surfaces under static conditions, provide oxygen and nutrient distribution with cells grown in flat layers that do not represent these cells’ physiological environment [2]. Moreover, 2D cultures have a relatively lower surface area to volume ratio, necessitating the use of a large number of dishes or flasks to attain the high cell density required for clinical applications. One of the methods for expanding MSCs is the use of microcarriers. In the microcarriers-based cultures, cells adhere to and proliferate on the surface of small beads suspended in a growth medium, allowing for scalable cultures due to increased surface area per volume. Various materials, such as polystyrene, gelatin, and alginate, have been developed to fabricate microcarriers with distinct physical properties [3]. MSCs cultured on gelatin methacrylate microcarriers have demonstrated a high stemness index with high proliferation ability [4]. This undifferentiated state was maintained through the regulation of cell-extracellular matrix (ECM) interactions, highlighting the advantages of microcarriers in providing a biomimetic environment conducive to MSC expansion. However, most cell-based therapies require a final product free of microcarriers or other microparticles to avoid safety concerns, which poses significant challenges for purification [3].

MSCs self-assemble under suspension conditions to form cell aggregates known as MSC spheroids. MSC spheroids create a specialized niche with tight cell-cell and cell-ECM interactions, thereby mimicking the in vivo environment more closely than conventional 2D culture methods [2]. MSC spheroids are expected to enhance stem cell properties such as differentiation [5, 6], angiogenic potential [7, 8], immunomodulatory effects [9, 10], and stemness [11, 12] while improving anti-apoptotic effects [13], thereby enhancing therapeutic efficacy by increasing cell transplantation efficiency. Microcarriers-based MSC cultures offer an efficient method for cell expansion; however, MSCs in spheroids reside in a quiescent nonproliferative state. This makes MSC spheroid cultures an effective priming method to prepare therapeutic cells [14]. MSC spheroids can be generated using either scaffold-based or scaffold-free methods. Scaffold-based techniques create multicellular 3D aggregates using different materials, offering advantages such as rapid fabrication and easy standardization of microsphere size [15,16]. For instance, MSC-collagen microspheres have been shown to enhance MSC chondrogenesis by mimicking the mesenchymal condensation stage, promoting cell-ECM interaction, and upregulating chondrogenic and osteogenic transcription factors [15]. Another study developed hybrid MSC spheroids incorporating methylcellulose and rapamycin-releasing poly (lactic-co-glycolic acid) microparticles (RAP-MPs), which had immunomodulatory effects and significantly improved islet survival in diabetic mice by increasing MSC expression of programmed death-ligand 1 [16]. In contrast, scaffold-free MSC spheroids are generated by the self-organization of cells under a non- or low-adherent environment, as described later. Despite numerous reports on suspension culture methods using MSCs, there are currently no standard protocols.

**Figure 1.**
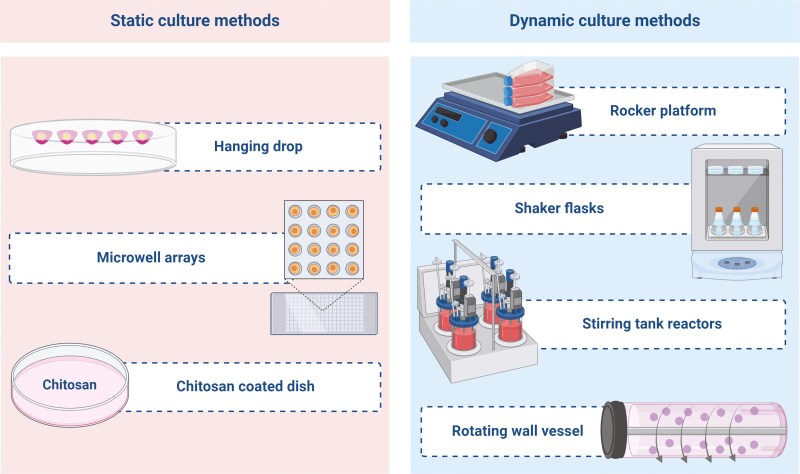
Representative culture method for static and dynamic culture of MSC spheroids. MSC spheroids from static culture are induced by cell aggregation using non- or ultralow-adhesive surfaces. In contrast, MSC spheroids from dynamic culture are fabricated under fluid flow. Created with BioRender.com/i14a164.

In response to these challenges, attention has shifted to the novel concept of culturing MSCs in suspended and floating environments. Floating culture systems can be broadly categorized into 2 main types: “static suspension cultures” and “dynamic suspension cultures” (Figure 1). Static suspension culture (Supplementary Table S1) involves seeding MSCs onto low-adhesion plates with microwells to allow cell aggregation and formation of cell clusters or spheroids over several hours to a few days [17]. Other methods, such as the use of chitosan membranes [12] and the hanging drop technique, [5] have also been employed in static culture systems. Static suspension culture offers simplicity by not requiring special equipment; however, it often poses technical challenges for media exchange, making it necessary to either use cell aggregates immediately after formation or transition to a dynamic suspension culture for maintenance. Dynamic suspension culture (Supplementary Table S2) involves culturing cells in a suspended environment with agitation or rotation, using methods such as rotation platforms [18] or shaker flasks [19]. Compared with static suspension cultures, controlling the size and number of cell aggregates is difficult in dynamic suspension cultures. However, it offers advantages in terms of nutrient and oxygen supply, enabling long-term cultivation [20,21]. Although it has been reported that certain types of stem cells are affected by shear stress in terms of cell functions such as multipotency and differentiation capacity [22,23], the effects of dynamic suspension culture environments on MSC spheroids remain unclear.

**Figure 2.**
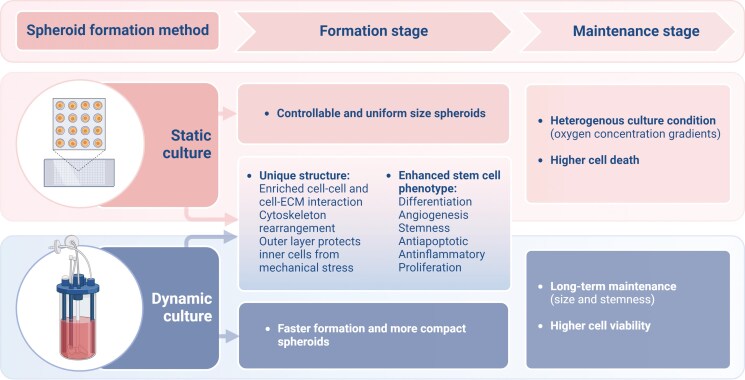
Schematic image indicating the difference between MSC spheroids cultured under static or dynamic suspension culture. Created with BioRender.com/d15z365.

A few studies have compared the behavior of MSC spheroids formed in static versus dynamic suspension cultures, particularly focusing on the notable differences in their agitation conditions. Therefore, in this study, we divided the MSC spheroid culture process into 2 distinct stages: the formation stage (development phase to form mature spheroids) and the maintenance stage (cultivation phase after the formation of mature spheroids). We then compared the outcomes to determine the differences between these cultivation methods under scaffold-free conditions (Figure 2). In particular, we discuss the advantages of dynamic suspension cultivation methods.

## MSC spheroids in the formation stage

### Cell aggregates and ECM production

The formation of the 3D-MSC spheroids consists of 3 steps. Initially, single floating cells bind to ECM proteins via integrins to form loose 3D structures. As cell-cell contact increases, cadherin expression increases, accumulating on the cell membrane surface. Finally, compact spheroids are formed via cadherin-cadherin binding [24]. The time required for cell aggregation varies for each cultivation method. In static suspension cultures, spheroids were formed within 12-24 hours using the microwell method [17, 25, 26] and within 24-48 hours using the hanging drop method [5, 7, 27]. Various studies have reported the formation of spheroids using dynamic suspension cultures. For instance, when umbilical cord-derived MSCs were cultured on a glass plate in a rocker system at 10 rpm, small spheroids were observed within 24 hours of cultivation, whereas shaking the flasks at 80 rpm resulted in spheroid formation overnight (12-24 hours) [6, 19]. Furthermore, spheroid formation has been achieved within 24-72 hours [8, 13, 21, 28]. Dynamic suspension cultures offer the possibility for modifying diverse conditions such as cell inoculation density, agitation rate, and agitation systems. Owing to the lack of established parameters for evaluating the formed spheroids and the absence of clear protocols for optimal culture conditions, the timing of spheroid formation varies compared with static suspension culture. In contrast, Park et al. reported the early acquisition of compact spheroids (within 12 hours) using an anti-gravity bioreactor with an acoustic levitation system, which outperformed conventional hanging drop methods [29]. This suggests that setting appropriate dynamic conditions may promote the formation of cell aggregates more effectively than in static suspension culture, thus potentially serving as an indicator for evaluating the timing of spheroid formation in future dynamic suspension cultures.

ECM is a critical factor in determining stem cell fate. The abundant ECM in MSC spheroids compared with 2D MSCs is implicated in the enhancement of MSC functions such as anti-apoptosis, enhanced proliferation, and increased paracrine effects [30]. Similar ECM expression was observed in MSC spheroids despite the different cultivation methods. MSC spheroids derived from adipose tissue formed by the hanging drop method showed increased expression of collagen I, fibronectin, and laminin, whereas MSC spheroids derived from the umbilical cord formed by the spinner flask method showed abundant expression of collagen I, fibronectin, laminin, and collagen IV [8, 31]. Furthermore, a study comparing MSC spheroids formed by static suspension culture (microwell plate) and dynamic suspension culture (stirring flask) by Allen et al. concluded that the difference in ECM generation between static and dynamic environments was less significant than the effects of cell signaling and oxygen/nutrient supply owing to spheroid size [26]. MSCs cultured on scaffolds promote the generation of ECM under mechanical force [32]; however, previous studies have not shown that dynamic cultivation environments are advantageous for ECM generation in MSC spheroids compared with static cultures. This may be due to the unique structure of MSC spheroids. Regardless of static or dynamic cultivation conditions, compact MSC spheroids consist of an outer dense and elongated cell layer and an inner round cell layer [5, 20]. It can be speculated that the dense outer cell layer in dynamic cultivation environments acts as a barrier, protecting the inner cells from cell death induced by shear stress while also blocking effective stimuli such as ECM generation.

### In vitro analysis of MSC spheroids

It is known from in vitro analyses that MSC spheroids exhibit improved properties compared with conventional 2D-cultured MSCs. However, the differences in the properties of spheroids formed under static and dynamic suspension culture conditions have not been elucidated.

MSC stemness was evaluated based on stem cell marker expression and clonogenicity. Studies have reported that spheroids formed under static [33, 34] and dynamic suspension culture [6, 11] exhibit higher expression of pluripotency marker genes, such as *octamer binding factor 4* (*Oct4*), *SRY-box transcription factor 2* (*Sox2*), and *Nanog* compared with 2D MSCs. Analysis of the clonogenicity of spheroid-derived single cells formed under different culture conditions also demonstrated a higher colony-forming ability than 2D MSCs [5, 11, 33]. Furthermore, studies examining differentiation potential have reported that both spheroids and spheroid-derived single cells have higher differentiation capacity than 2D MSCs [17, 19, 33]. When comparing the expression of MSC markers, it was shown that while MSC spheroids maintained properties characteristic of MSCs compared with 2D MSCs, the expression of CD90 and CD105, which are representative markers of MSCs, decreased in MSC spheroids [5, 8, 35]. CD90 plays an important role in the fate of MSCs by regulating their differentiation toward adipocytes or osteoblasts [36]. Wang et al. suggested that the reduction of CD90 in MSC spheroids may indicate a sensitive form of undifferentiated cells that can rapidly differentiate by targeted signal transduction [37]. Similarly, CD105, a high-affinity coreceptor for transforming growth factor (TGF)-β, is known to regulate the MSC differentiation toward osteoblasts and chondrocytes [38, 39]. However, the influence of decreased CD90 and CD105 expression on the function of MSC spheroids remains unclear. Decreased expression was observed in both static and dynamic culture-derived MSC spheroids, and the expression recovered when MSC spheroids were plated back into a 2D environment, suggesting that the downregulation of these molecules was related to the 3D structure of MSC spheroids rather than to culture conditions.

### Advantages of the 3D structure of MSC spheroids

The importance of MSC’s 3D structure is discussed not only in terms of ECM composition but also in terms of changes in cellular structure. E-cadherin, a key adhesion molecule of the cadherin family, plays a crucial role in mediating cell-cell adhesion in MSC spheroids [11, 40]. Inhibiting E-cadherin disrupts the spheroid formation, while its activation enhances both the proliferative and paracrine potential of MSCs through extracellular signal-regulated kinase/v-akt murin thymoma viral oncogene homolog1 (ERK/AKT) signaling pathways [40]. Furthermore, inhibition of Rho/ROCK signaling, a major regulator of cytoskeletal contraction, which mediates cellular migration and cell-cell adhesion, significantly disturbed spheroid formation and decreased stemness gene expression [12]. These results suggested a crucial role of upregulated adhesion molecules and cytoskeletal rearrangement in MSC spheroid formation to enhance their stem cell phenotype. Dissociated single cells derived from MSC spheroids are smaller than those derived from 2D MSCs [5,8]. Smaller cells in spheroids are composed of thin parallel actin filaments and exhibit lower cytoskeleton tension than the well-spread actin filaments observed in 2D MSCs [41]. Therefore, Zhou et al. focused on the relationship between cytoskeletal tension and MSC stemness. The authors demonstrated that releasing cytoskeletal tension in 2D MSCs using cytochalasin D led to increased *Nanog* expression, suggesting that the cellular structure of MSC spheroids contributes to the enhancement of MSC properties. Additionally, dramatic cytoskeletal reorganization due to MSC aggregation alters the morphology of intracellular organelles such as mitochondria, leading to changes in metabolic configuration and enhancement of MSC stemness [42]. The direct comparative studies of MSC spheroids’ structure cultured by static or dynamic suspension have not yet been conducted. However, the drastic structural change from 2D to 3D MSCs seems to have a greater influence on MSC function, which was implied by the comparable structure and function of MSC spheroids formed by different suspension culture methods.

Three-dimensional constructs exhibit oxygen gradients leading to oxygen limitation in the core of MSC spheroids, which naturally creates a hypoxic environment. MSCs mainly respond to hypoxia by the stabilization of hypoxia-inducible factor 1α (HIF-1α), triggering oxygen-dependent regulatory pathways that influence their metabolic fate and multipotency [43]. Stabilization of HIF-1α in MSCs is known to induce pluripotent gene expression and promote increased proliferation [44]. Compared with 2D MSCs, MSC spheroids showed higher expression of HIF-1α and hypoxia-induced survival factors, such as hepatocyte growth factor (HGF), vascular endothelial growth factor (VEGF), fibroblast growth factor 2 (FGF2), and stromal cell-derived factor [13,45], with their expression levels increasing with culture duration and spheroid size. Moreover, the influence of spheroid formation methods on the onset of hypoxia in MSC spheroids was discussed in the previous studies. Schimitz et al. fabricated MSC spheroids by ultra-low attachment dish or hanging drop system and monitored hypoxia by detecting fluorescent-labeled hypoxia reporter proteins [46]. Despite similar spheroid sizes, between low attachment dish- and hanging drop-fabricated spheroids, low attachment dish-fabricated spheroids showed remarkably higher fluorescent intensity. This difference was attributed to better oxygen supply in hanging drops due to smaller medium volumes and larger gas-liquid interface which delayed the onset of hypoxia in MSC spheroids. Notably, the authors highlighted that the microwell plates showed unequal oxygen distribution with concentrations decreasing toward the edges. This limitation of static suspension culture may result in biologically unequal spheroids. In contrast, dynamic suspension cultures, which involves gentle mixing of fluids, provide a more homogeneous culture environment [47]. MSC spheroids generated under shaker flasks showed increased expression of HIF-1α and enhanced secretion of angiogenic and anti-apoptotic factors [13]. These results suggested that dynamic suspension cultures may offer a more biologically uniform spheroid culture environment than static methods, ensuring a more consistent oxygen and nutrient supply.

### Rejuvenation of MSC spheroids

In recent years, there has been a focus on the relationship between changes in the cellular structure of MSC spheroids and MSC rejuvenation. MSCs cultured for prolonged periods under adherent conditions undergo changes in gene and protein expression as well as a decline in differentiation potential, ultimately showing replicative senescence. However, spheroid formation from aged MSCs results in a reduction in aging-related marker expression, recovery of differentiation potential, upregulation of stem cell markers, and an increase in immunomodulatory factors, indicating cellular rejuvenation [14,20,21,48]. Surprisingly, the transcriptional profiles of MSC spheroids derived from both young and aged MSCs were remarkably similar, showing greater resemblance to each other than to their 2D MSC counterparts [14]. MSCs cultured for long periods under adherent conditions exhibit cellular hypertrophy and irregularly distributed actin filaments. However, through 3D formation of expanded MSCs, dramatic cytoskeletal rearrangement occurs, and spheroid-derived cells re-adopt a regular arrangement of actin with clear leading and trailing edges, similar to that of pre-aging MSCs [48]. Krasnova et al. proved that the rejuvenation effects were not due to a negative selection of senescent cells by observing the incorporation and morphological alternation of senescent cells in spheroids [48]. While the mechanism of this rejuvenation is not yet fully understood, it has been emphasized that the 3D structure of MSCs is an important factor potentially associated with drastic changes in actin cytoskeleton which lead to changes in the Golgi apparatus structure and activated autophagy capacity [48]. These novel insights demonstrate that 3D culturing of MSCs can be an effective method to overcome replicative senescence, a major challenge in the therapeutic use of MSCs.

## MSC spheroids in maintenance stage: long-term maintenance of spheroids

Long-term culture of MSC spheroids suggested that static and dynamic suspension cultures have different effects on MSCs. Wolff et al. observed that human adipose-derived MSCs cultured for 1 month under static suspension conditions did not exhibit an apparent necrotic core due to cell death but showed a continuous decrease in cell numbers [49]. Bellotti et al. cultured spheroids formed from human bone marrow (BM)-derived MSCs under static suspension conditions and observed that while the spheroids remained morphologically homogeneous until the first month, necrosis of the internal cells was observed in the second month [50]. This decrease in cell number was also evident as a reduction in spheroid diameter; Wolff et al. reported a significant decrease in size from an initial diameter of 960-560 µm after 7 days of culture, while Bellotti et al. reported a decrease from ~900 to ~600 µm after 21 days of culture. In the hanging drop method of static suspension culture, an increase in apoptotic or necrotic cells was observed with the extension of the culture period from the 3rd to 4th day, suggesting that cell death increased as the spheroid size increased [5].

In contrast, the spheroids formed under dynamic suspension culture conditions exhibited different behaviors. In our study, despite forming large spheroids of 1200 µm in size using shaking flasks for dynamic suspension culture, the cell number and spheroid size remained unchanged and sustained over a long-term culture period of 1-3 weeks [21]. Furthermore, successful maintenance of MSC spheroids for an extended period of 2 months in shaking flasks has been achieved, demonstrating the preservation of both morphology and MSC-related marker expression and multipotency [20].

The maintenance of stemness in MSC spheroids is believed to be associated with the quiescent state of MSCs. Stem cells exist in a quiescent state in vivo and exhibit rapid responsiveness to various signals to perform stem cell functions [51]. Although MSC spheroids initially show active cell proliferation, characterized by expression of high proliferating cell nuclear antigen [29], prolonged culture leads to a gradual decrease in the expression of proliferation markers such as Ki67 and BrdU, resulting in suppressed cell proliferation [8, 17]. MicroRNAs (miRNAs) crucial for maintaining the quiescent state of adult stem cells, such as miR-489, miR-370, and miR-433, are highly expressed in MSC spheroids [34]. Additionally, compared with 2D MSCs, cells constituting spheroids exhibit a predominance of cells in the G0/G1 stage of the cell cycle, indicating a quiescent state where active cell proliferation is inhibited and metabolism is decreased, thereby exerting a protective effect on cells [52].

When spheroids in a quiescent state adhere to culture plates in 2D cultures, vigorous cell proliferation is observed [17, 20, 48]. These results suggest that MSC spheroids maintain favorable cellular properties over long-term culture in a quiescent state. However, as mentioned earlier, compared with dynamic suspension culture, cell death increases in static suspension culture over time. The apoptosis/necrosis of cells in spheroids occurs by limited oxygen diffusion and metabolic consumption in the core of cultured spheroids, which are strongly influenced by culture conditions [47]. Anada et al. showed that using a gas-permeable device to generate tumor cell aggregates inhibited hypoxia-induced necrosis, allowing for sustained cell growth and metabolic activity [53]. A hypoxic core was observed in MSC spheroids with >700 μm in the low attachment dish and in MSC spheroids with >900 μm in the hanging drop [46]. This difference between different culture methods occurred due to the different medium volumes and gas-liquid interface allowing the hanging drop system to provide a better oxygen supply. Moreover, dynamic suspension culture, facilitated by fluid flow and agitation of the medium, provides a uniform supply of nutrients and oxygen as well as the removal of metabolic waste, enhancing cell viability and proliferation [54]. Studies culturing pre-osteoblasts on scaffolds under static or dynamic conditions have reported improved cell viability in the center of the scaffold under dynamic conditions owing to the advantageous oxygen supply, demonstrating the advantages of dynamic suspension culture in decreasing cell death [55].

The underlying mechanism of the long-term maintenance of MSC spheroids is still under investigation. However, recent studies have shown the advantages of dynamic cultivation of MSC spheroids in delivering a steady supply of functional MSCs.

## Potentials of dynamic culture MSC spheroids in regenerative medicine

**Figure 3.**
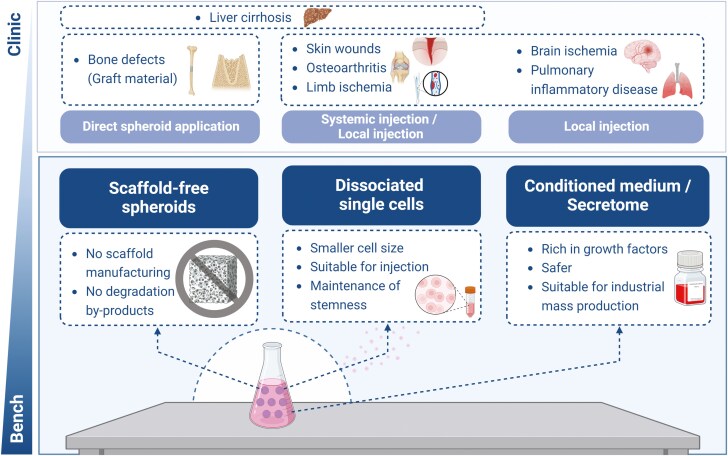
Therapeutic applications of dynamic MSC spheroids. Created with BioRender.com/c75t568.

The vast majority of reported animal studies and clinical trials have consistently highlighted the superior regenerative abilities and therapeutic potential of MSC spheroids (Supplementary Table S3). These attributes are presumed to be more pronounced in spheroids generated through dynamic suspension cultures, as discussed earlier. Such advancements offer promising treatment avenues for debilitating conditions, such as osteoarthritis, pulmonary inflammatory diseases, liver cirrhosis, severe skin wounds, and brain and limb ischemia [9, 13, 28, 56-58] (Figure 3). However, the lack of standardized conditions and protocols for MSC spheroid fabrication, coupled with the challenge of balancing therapeutic efficacy, safety, and cost-effectiveness, impedes the generalization of existing in vitro and in vivo findings and their translation into clinical applications [59].

The scaffold-free structure of MSC spheroids, particularly those produced via dynamic suspension culture, presents another significant advantage for clinical applications. Unlike scaffold-based methods, the enhanced cell-cell contact and cell-ECM interactions in the free structure of MSC spheroids also reduce the likelihood of immune reactions toward the scaffold materials. Additionally, it mitigates the technical complexities associated with cell seeding on scaffolds and the potential adverse effects resulting from cell interactions with scaffold materials or their degradation by-products [59, 60].

The utilization of MSC spheroids as bone graft material is inevitable because of their exceptional osteogenic regenerative capacity. Suenaga et al. transplanted human MSC spheroids, which were induced to form osteogenic spheroids, into rat calvarial defects. They reported that applying MSC spheroids alone as a grafting material resulted in higher and more uniform bone formation in comparison with β-TCP and even a combination of MSC spheroids and β-TCP [60]. Furthermore, the spheroid-only group exhibited better manipulation and adherence to the defect walls, highlighting the advantage of the scaffold-free structure of the spheroids, which relies on self-secreted ECM. Similarly, our group investigated the osteogenic regenerative capacity of dynamic suspension culture-generated serum-free neural crest medium (NM) spheroids maintained for 3 weeks [21]. In comparison to 2D adherent BM-MSCs, and even conventional growth media spheroids, the application of NM spheroids to rat femur defects resulted in a thicker and more highly mineralized layer of bone with almost complete closure of the defects. These findings underscore the successful maintenance of therapeutic MSC spheroids during prolonged cultivation through dynamic suspension culture and highlight the significance of culture media in the properties of the resulting spheroids [21].

While the direct application of 3D MSC spheroids may not be the ideal method of administration for certain defects or diseases, intravenous (IV) injection of single cells detached from the spheroids, instead of applying the spheroids themselves, could offer a promising alternative approach because of the superior homing ability of spheroid-derived MSCs to target wounded tissues. This can be attributed to the decreased expression of integrins in 3D MSCs in comparison to 2D MSCs. This reduces the risk of entrapment in the lung and improves distribution to injured tissues. Additionally, 3D culture restored the expression of homing receptors such as CXCR4, enhancing the distribution of MSCs in the injured area. Moreover, MSCs from 3D spheroids were significantly smaller than those from 2D cultures, reducing the possibility of vascular obstructions in the lungs after IV injection [56,61]. Notably, MSC spheroids cultured under dynamic suspension culture successfully reattached to the cell culture dish when reseeded in a static condition and produced migratory cells while maintaining their roundness and size even at day 7 [20]. Moreover, to investigate if they would sustain their proliferative capacity, these spheroids were repeatedly harvested and reseeded 4 more times and continued to provide migratory cells in abundance. Considering their ability to maintain multipotency, stemness, and immunomodulatory properties, along with their capacity to yield sufficient cell numbers even after multiple reseeding, MSCs derived from dynamic suspension culture present a cost-effective and efficient therapeutic approach.

Using the conditioned medium (CM) of MSC spheroids rather than spheroids or detached cells presents another intriguing potential application, as numerous studies have indicated that the secretome of MSC spheroids is as significant and efficient as that of the spheroids themselves [8, 9, 59]. Compared with CM from 2D culture, CM from 3D dynamic suspension culture has been reported to contain significantly higher levels of proangiogenic factors such as VEGF, FGF2, HGF, CXCL2, and CXCL12 [8, 13, 62], alongside increased secretion of some immunomodulatory factors such as TGF-β, interleukin (IL)-10, LIF, and IL-6 [8, 9]. The dynamic suspension culture of MSC spheroids provides a more homogenous environment than monolayer expansion and static suspension culture, allowing extended culture of spheroids for up to 2 months [20]. The secretome of MSC spheroids tends to increase in time-dependent and spheroid-size-dependent manner; therefore, CM obtained from dynamic suspension culture may result in a high-quality CM rich in growth factors and cytokines [13, 45, 59]. This potency can be further enhanced by tailoring spheroid culture conditions to produce specific growth factors or increasing paracrine properties based on the target tissue or patient conditions, a process known as priming or licensing in case of immunomodulatory applications. One study, using a rotary orbital shaker, primed human MSC spheroids with TNF-α and IFN-γ, which led to increased IDO activity, higher IL-6 secretion, and enhanced suppression of macrophage TNF-α secretion compared with untreated spheroids [63]. This CM-based approach offers several advantages over traditional cell therapy, including easier storage, transportation, and the elimination of cell transplantation risks, making it suitable as a “ready-to-go” biological product [8, 9, 59]. In addition to the biological advantages of dynamic suspension culture over static suspension culture for CM production, it has an additional advantage from an industrial viewpoint as it facilitates easy exchange and collection of media without disturbing the formation of spheroids [20]. Moreover, the physical factors such as oxygen tension, shear stress, and temperature, which can be standardized and uniformly distributed in the dynamic suspension cultures, can further enhance the quality of CM, unlike in 2D and static 3D cultures [59]. This benefit in terms of CM mass production enables continuous standardized manufacturing of high-quality CM at a lower cost, which is required for large-scale therapeutic applications [59].

## Application strategy of dynamic culture MSC spheroids

Generating clinical-grade MSC spheroids can be time-consuming, especially when autologous cells are used. This emphasizes the importance of cryopreserving MSC spheroids for prospective use in regenerative applications, drug testing, and personalized treatments, which can be life-saving for patients with life-threatening medical conditions who cannot afford delays in spheroid preparation. Cryopreservation, however, can impact cell viability and proliferation, and cause cell loss upon recovery [64]. Arai et al. reported that while collagen type I expression and cell proliferation remained stable, the viability of cryopreserved fibroblast-based spheroids decreased, which was strongly affected by the type and concentration of the cryoprotectant solution used. They also discussed the importance of washing spheroids after thawing, as the cryoprotectant solution can cause cytotoxic effects, if not properly removed. In single-cell suspensions, cryoprotectants are typically washed out, but in spheroids, residual cryoprotectant inside the structure led to cell death, which could be mitigated by additional washing steps.

Jeong et al. [65] tried to enhance the cell viability of the MSC spheroids after thawing by using techniques such as vitrification with a higher concentration of the cryoprotectant solution instead of slow freezing which entailed higher cell viability across various sizes (200-900 µm). They speculated that a high concentration of the cryoprotectant solution permeates the core region of the spheroids more effectively, which protects the cells from freezing damage. Similarly, Motoike et al. [66] revealed the cryoprotective properties of ECM, especially type Ⅰ collagen, in MSC spheroids. They observed comparable MSC marker expressions and bone regeneration ability of cryopreserved ECM-abundant MSC spheroids to those of noncryopreserved spheroids, in contrast with the cell collapse observed by cryopreserved ECM-sparse spheroids. These results were associated with anoikis, a specific type of cell apoptosis induced by loss of cell-cell of cell-ECM contacts, suggesting the importance of the 3D component of MSC spheroids on cryopreservation. Despite these advances, there is no consensus on an optimal cryopreservation protocol. The effects of cryopreservation on various cell types and markers remain to be fully explored, and a standardized, long-term cryopreservation protocol for MSC spheroids is needed to maximize their benefits and ensure safe biobanking.

Establishing cost-effective Good Manufacturing Practice (GMP)-compliant biofabrication protocols for MSC spheroids is essential for their future clinical translation. To achieve clinical-grade spheroids detailed product specifications are necessary, including comprehensive biomolecular analyses such as liquid chromatography-mass spectrometry, and bioinformatic analysis [67]. This enables the development of a quality control (QC) framework that addresses safety, identity, potency, sterility, proliferative capacity, purity, and viability, ensuring high predictability and reproducibility across batches [67, 68]. While static MSC spheroids can be successfully produced on a large scale with a quality that fulfils the GMP requirements, dynamic MSC spheroids offer greater potential to meet various aspects of the QC standards [59, 67]. Unlike static MSC spheroids, dynamic systems allow for the simultaneous production of 3 therapeutic products: the spheroids themselves, the collection of CM, and the harvest of large numbers of single detached cells through reseeding [20,21,59].

Large-scale dynamic culture systems equipped with sensors enable real-time monitoring and control of key operational variables such as oxygen levels, pH, nutrients, byproducts, and growth factor levels, creating a more homogeneous microenvironment. This automation facilitates high sterility and reproducibility of the resulting products, in line with the risk assessments and QC standards [59]. Additionally, dynamic cultures offer greater flexibility for preconditioning and manipulation. For instance, inducing osteogenic differentiation, which is more easily achieved compared with static 3D cultures, the spheroids are more prone to disruption [59,67].

## Conclusion

MSC spheroids formed by static and dynamic suspension cultures exhibit a unique structure and an enhanced stem cell phenotype. Recent evidence highlighted that dynamic culture environments during the formation stage stimulate MSCs to produce growth factors and cytokines, resulting in faster and more compact spheroid formation compared with static suspension culture. Additionally, the long-term maintenance of MSC spheroids under dynamic conditions maintains their size without central cell death and sustains the expression of stemness-related genes. The dense outer layer of MSC spheroids in dynamic culture environments appears to act as a barrier, protecting the inner cells from cell death induced by shear stress while blocking signal transduction that could lead to spontaneous differentiation.

The unique characteristics of MSC spheroids, particularly produced by dynamic suspension culture, provide promising tools for the therapeutic use of stem cells, including scaffold-free spheroids as bone graft material, spheroid-derived single cells for intravenous injection, and spheroid-derived secretomes. Further research on directly comparing MSC spheroids cultured under static and dynamic conditions, as well as in vivo experiments, is needed to better understand the mechanisms and biological outcomes of MSC spheroids for future therapeutic applications in various diseases and regenerative medicine.

## Supplementary material

Supplementary material is available at *Stem Cells Translational Medicine* online.

szae093_suppl_Supplementary_Table_S1

szae093_suppl_Supplementary_Table_S2

szae093_suppl_Supplementary_Table_S3

## Data Availability

No new data were generated or analyzed in support of this research.
