# Supplementary material for: Current perspectives on the dynamic culture of mesenchymal stromal/stem cell spheroids
Source: Stem Cells Transl Med. 2024 Dec 31;14(3):szae093. doi: 10.1093/stcltm/szae093 (PMC11954588; doi:10.1093/stcltm/szae093)
Supplement: szae093_suppl_Supplementary_Table_S1 [file szae093_suppl_supplementary_table_s1.docx]

**Supplementary Table 1.** **List of *in vitro* studies on MSC spheroid formation using static culture**

| References  [reference number] | Cell type | Method | Culture duration  (spheroid formed) | Medium | Initial cell number |
| --- | --- | --- | --- | --- | --- |
| Bartosh TJ *et al*. *Proc Natl Acad Sci U S A* 2010;107:13724–13729  [5] | Human  BM-MSCs | Hanging drop | 4 days  (48–96 h) | αMEM, 17% FBS, 100 units/mL penicillin, 100 μg/mL streptomycin, 2 mM L-glutamine | 35 μL drops of medium containing 1 × 10^4^ –2.5 × 10^5^ cells |
| Huang GS *et al*. *Biomaterials* 2011;32:6929–6945  [12] | Human  AD-MSCs | Chitosan or chitosan-hyaluronan (HA) membranes | 10 days  (chitoan 41 h, chitosa-HA 13 h) | DMEM-LG/F12 (1:1), 10% FBS, 10 mg/L P/S, 10mg/L L-glutamine | 3 × 10^4^ cells/well in 24-well plates |
| Hildebrandt C *et al*. *Tissue Cell* 2011;43:91–100  [18] | Human  BM-MSCs | Hanging drop | 2 days  (NA) | αMEM, 15% FBS, 100 units/ml penicillin, 100 μg/mL streptomycin | 20 μL drops of medium containing 5 × 10^3^ cells |
| Ylöstalo JH *et al*. *Stem Cells*  2012;30:2283–2296 | Human  BM-MSCs | Hanging drop | 3 days  (NA) | αMEM, 17% FBS, 100 units/mL penicillin, 100 μg/mL streptomycin, 2 mM L-glutamine | 35 μL drops of medium containing 2.5 × 10^4^ cells |
| Cheng NC *et al*. *Biomaterials* 2012;33:1748–1758  [33] | Human  AD-MSCs | Chitosan membranes | 14 days  (24 h) | DMEM-HG, 10% FBS, 1% P/S | 1.5 × 10^3^–5 × 10^4^ cells/cm^2^ |
| Cheng NC *et al*. *Stem Cells Transl Med* 2013;2:584–594 | Human  AD-MSCs | Chitosan membranes | 14 days  (NA) | DMEM- HG, 10% FBS, 1% antibiotic-antimycotic | 2.5 × 10^4^ cells/cm^2^ |
| Alimperti S *et al*. *Biotechnol Prog* 2014;30:974–983  [19] | Human  BM-MSCs | Non-adherent plate | 5 days  (NA) | Serum-free medium | 5 × 10^4^–1 × 10^5^ cells/mL |
| Guo L *et al*. *J Cell Mol Med* 2014;18:2009–2019  [34] | Human  AM-MSCs | Hanging drop | 100 h  (36 h) | DMEM, 0-30% FBS, antibiotics | 35 μL drops of medium containing 3 × 10^2^–3 × 10^5^ cells |
| Xu Y *et al*. *J Cell Mol Med*  2016;20:1203–1213  [31] | Human  AD-MSCs | Hanging drop | 3 days  (NA) | αMEM, 17% FBS, 100 units/mL penicillin, 100 μg/mL streptomycin, 2 mM L-glutamine | 35 μL drops of medium containing 2.5 × 10^4^ cells |
| Cesarz Z *et al*. *Stem Cells Dev* 2016;25:622–635 | Human  BM-MSCs | Hanging drop | 4 days  (NA) | αMEM, 17% FBS, 2 mM L-glutamine, 1 mM pyruvate, 100 μM nonessential amino acids, 100 units/mL P/S | 10 μL drops of medium containing 1 × 10^4^ cells |
| Bellotti C *et al. Cytotechnology*  2016;68:2479–2490  [50] | Human  BM-MSCs | 1.5 mL polypropylene conical tubes | 2 months  (3 days) | DMEM-HG, 10% FBS | 2.5 × 10^5^ cells/tube |
| Cho RJ *et al*. *BMB Rep*  2017;50:79–84 | Human  AD-MSCs | Polydimethylsiloxane (PDMS)- based concave microwells | 1 day  (1 day) | MesenPRO RS Medium, Growth Supplement, 1% penicillin | 1 × 10^5^ cells/well |
| Zhou Y *et al*. *J Cell Mol Med*  2017;21:1073–1084  [41] | Human  AM-MSCs | Hanging drop | 60 h  (NA) | DMEM, 10% FBS | 35 μL drops of medium containing 2 × 10^4^ cells |
| Liu Y *et al. Stem Cells*  2017;35:398–410  [42] | Human  BM-MSCs | Non-adherent plates | 3 days  (10 h) | αMEM, 10% FBS, 1% P/S | 1.5 × 10^3^ cells/cm^2^ |
| Jiang B *et al. Biomaterials*  2017;133:275–286  [52] | Human  BM-MSCs | Hanging drop | Overnight  (NA) | MSC medium | 25 μL drops of medium containing 2.5 × 10^4^ cells |
| Miceli V *et al*. *Stem Cells Int* 2019; 2019:7486279  [10] | Human  AM-MSCs | 6 well non-adhesive plates | 3 days  (1 day) | DMEM serum-free medium | 5 × 10^5^ cells/mL |
| Bartosh TJ *et al.* *Cells*  2019;8:1031  [14] | Human  BM-MSCs | Hanging drop | 3 days  (NA) | Complete culture medium: αMEM, 17% FBS, 100 units/mL penicillin, 100 μg/mL streptomycin, 2 mM L-glutamine  Xenofree medium: StemPro XF, 13 mg/mL human serum albumin | 35 μL drops of medium containing 2.5 × 10^4^ cells |
| Allen LM *et al*. *Stem Cells Int* 2019;2019:4607461  [26] | Human  Syf-MSCs | Aggrewell 400 microwell plates (STEMCELL) | 12 days  (1 day) | Serum-free medium | 5 × 10^2^ –2 × 10^4^ cells/microwell |
| Schmitz C *et al. Front Bioeng Biotechnol*  2021; 9:611837  [46] | Human  AD-MSCs | 96 well non-adherent plates or hanging drop | 1 day  (1 day) | αMEM, 1 g/L glucose, 2 mM L-glutamine, 10% human serum, 50 μg/mL gentamicin | 1 × 10^4^–3 × 10^5^ cells/spheroid |
| Wu DT *et al*. *Bioeng Transl Med* 2022;8:e10464 | Mouse/Human  BM-MSCs | Aggrewell 400 microwell plates (STEMCELL) | Overnight  (NA) | Mouse MSCs: GlutaMAX DMEM-HG, 10% FBS, 1% P/S  Human MSCs: MSC basal medium, 7% FBS, 15 ng/mL rh IGF-1, 125 pg/mL rh FGF-b, 2.4 mM L-alanyl-L-hlutamine, 1% P/S | Mouse MSCs: 1000 cells/spheroids  Human MSCs: 500 cells/spheroid |
| Krasnova O *et al. Stem Cell Res Ther*  2023;14:373  [48] | Human  AD-MSCs | Hanging drop | 3 days  (NA) | DMEM-LG, 10% FBS | 25 μL drops of medium containing 7 × 10^3^ cells |
| Wolff A *et al. Biomedicines*  2023;11:1049  [49] | Human  AD-MSCs | 96 well non-adherent plates | 35 days  (overnight) | Osteogenic medium: DMEM, 10% FBS, 1% P/S, 0.25 g/L ascorbic acid, 10 μM dexamethasone, 10 μM β-glycerophosphate | 1 × 10^5^ cells/well |

**Abbreviations:** AD-MSCs (adipose tissue-derived mesenchymal stromal/stem cells), AM-MSCs (amnion membrane-derived mesenchymal stromal/stem cells), BM-MSCs (bone marrow-derived mesenchymal stromal/stem cells), Syf-MSCs (synovial fluid-derived mesenchymal stromal/stem cells), UC-MSCs (umbilical cord-derived mesenchymal stromal/stem cells), DMEM-LG (DMEM-low glucose), DMEM-HG (DMEM-high glucose), P/S (penicillin-streptomycin), NA (not applicable)
