# Supplementary material for: Current perspectives on the dynamic culture of mesenchymal stromal/stem cell spheroids
Source: Stem Cells Transl Med. 2024 Dec 31;14(3):szae093. doi: 10.1093/stcltm/szae093 (PMC11954588; doi:10.1093/stcltm/szae093)
Supplement: szae093_suppl_Supplementary_Table_S2 [file szae093_suppl_supplementary_table_s2.docx]

**Supplementary Table 2. *In vitro* studies on MSC spheroid formation using dynamic or static/dynamic culture**

| References  [reference number] | Method | Cell type | Static culture/Initial cell number | Static culture duration  (spheroid formed) | Dynamic culture (agitation speed)/Initial cell number | Dynamic culture duration  (spheroid formed) | Medium |
| --- | --- | --- | --- | --- | --- | --- | --- |
| Frith JE *et al*. *Tissue Eng Part C Methods* 2010;16:735–749  [35] | Static＋Dynamic | Human  BM-MSCs | Nonadherent plates/1 × 10^6^ cells/mL | 6 h  (6 h) | Spinner flasks (30 rpm) or RWV (15 rpm)/2 × 10^4^ cells/mL | 7 days  (NA) | αMEM, 100 units/mL penicillin, 100 mg/mL streptomycin, 15% FBS |
| Cho YJ *et al*. *J Neurosci Res* 2012;90:1794–1802  [7] | Static＋Dynamic | Human  AD-MSCs | Hanging drop/30 μl drop of medium containing 1 × 10^6^ cells/mL | 1 day  (1 day) | Spinner flasks (70 rpm)/6 × 10^5^ cells/mL | 2 days  (NA) | αMEM, 10% FBS, 100 units/mL penicillin, 100 mg/mL streptomycin |
| Baraniak PR *et al*. *Cell Tissue Res* 2012;347:701–711  [17] | Static＋Dynamic | Mouse  BM-MSCs | Aggrewell 400 microwell plates (STEMCELL) /1.8-6 × 10^6^ cells/mL | 18 h  (18 h) | Rotary orbital shaker (45 ± 2 rpm)/~1500 spheres in 10 mL medium | 21 days  (NA) | IMDM, 10% FBS, 10% horse serum, 2 mM L-glutamine, 100 units/mL penicillin, 100 μg/mL streptomycin, 0.25 μg/mL amphotericin B |
| Bhang SH *et al*. *Tissue Eng Part A* 2012;18:2138–2147  [27] | Static＋Dynamic | Human  UC-MSCs | Hanging drop/30 μl drop of medium containing 1 × 10^6^ cells/mL | 1 day  (1 day) | Spinner flasks (70 rpm)/6 × 10^5^ cells/mL | 2 days  (NA) | αMEM, 10% FBS |
| Zimmermann JA *et al*. *Cytotherapy* 2014;16:331–345  [63] | Static＋Dynamic | Human  BM-MSCs | 400-µm agarose microwell plates/1.2-6 × 10^6^ cells/well in 6 well plates | 18 h  (18 h) | Rotary orbital shaker (65 rpm)/3 × 10^4^ cells/mL | 4 days  (NA) | αMEM, 16.5% FBS, 2 mmol/l L-glutamine, 100 units/mL penicillin, 100 mg/mL streptomycin or MesenCult-XF medium |
| Bhang SH *et al*. *Mol Ther* 2014;22:862–872 | Static＋Dynamic | Human  AD-MSCs | Hanging drop/30 μl drop of medium containing 1 × 10^6^ cells/mL | 1 day  (1 day) | Spinner flasks (45 rpm)/6 × 10^5^ cells/mL | 2 days  (NA) | αMEM, 10% FBS, 100 units/mL penicillin, 100 mg/mL streptomycin or clinically relevant medium |
| Costa MHG *et al*. *J Biotechnol* 2017;262:28–39 | Static＋Dynamic | Human  BM-MSCs | 400-µm agarose microwell plates/6 × 10^5^ cells/well in 24 well plates | 18 h  (18 h) | Rotary orbital shaker (65 rpm)/5 × 10^4^ cells/mL | 7 days  (NA) | DMEM-LG, 10% FBS, 1% antibiotic-antimycotic |
| Cha JM *et al*. *Sci Rep*  2018;8:1171  [25] | Static＋Dynamic | Human  BM-MSCs | Polyethylene glycol (PEG) hydrogel microwell arrays/5 × 10^5^ cells/array | 1 day  (12 h) | Rotary orbital shaker (30 rpm)/NA | 7 days  (NA) | DMEM-LG, 10% FBS or exosome-free FBS, 1% antibiotics-antimycotics |
| Allen LM *et al*. *Stem Cells Int* 2019;2019:4607461  [26] | Static + Dynamic | Human  Syf-MSCs | Aggrewell 400 microwell plates (STEMCELL)/5 × 10^2^ cells/microwell | 1 day  (1 day) | Spinner flasks (70 rpm)/5 × 10^4^ cells/mL | 12 days  (NA) | Serum-free medium |
| Bhang SH *et al*. *Biomaterials* 2011;32:2734–2747  [13] | Dynamic | Human  AD-MSCs | NA | NA | Spinner flasks (70 rpm)/6 × 10^5^ cells/mL | 3 days  (1 day) | αMEM, 10% FBS, 100 units/mL penicillin, 100 μg/mL streptomycin |
| Hildebrandt C *et al*. *Tissue Cell* 2011;43:91–100  [18] | Dynamic | Human  BM-MSCs | NA | NA | 96 well non adhesive plates on rotation platform (75 rpm)/1-2 × 10^4^ cells/well | 21 days  (2 days) | αMEM, 15% FBS, 100 units/mL penicillin, 100 μg/mL streptomycin |
| Alimperti S *et al*. *Biotechnol Prog* 2014;30:974–983  [19] | Dynamic | Human  BM-MSCs | NA | NA | Shaking flasks (80 rpm)/5 × 10^5^ cells/mL | 7 days  (1 day) | Serum-free medium |
| Li Y *et al*. *Cell Tissue Res* 2015;360:297–307  [6] | Dynamic | Human  UC-MSCs | NA | NA | Rocker system (10 rpm)/1 × 10^6^ cells/mL | 9 days  (24 h) | serum-free medium |
| Santos JM *et al*. *Stem Cell Res Ther*  2015;6:90  [8] | Dynamic | Human  UC-MSCs | NA | NA | Spinner flasks (80 rpm for formation, 110 rpm for maintanance)/1 × 10^6^ cells/mL | 11 days  (2 days) | αMEM, 2mM L-glutamine, 1 g/l glucose, 2.2 g/l sodium bicarbonate, 10% FBS |
| Zhang S *et al*. *Biomaterials* 2015;41:15–25  [11] | Dynamic | Human  AD-MSCs | NA | NA | Rotating wall vessel (25 rpm)/1 × 10^6^ cells/mL | 5 days  (24 h) | DMEM/F12, 1% P/S |
| Kwon SH *et al*. *J Surg Res* 2015;194:8–17  [62] | Dynamic | Human  AD-MSCs | NA | NA | Spinner flasks (70 rpm)/1 × 10^6^ cells/mL | 3 days  (NA) | αMEM, 10% FBS, 100 units/mL penicillin, 100 µg/mL streptomycin |
| He H *et al*. *Cell Prolif* 2019;52:e12587 | Dynamic | Rabbit  BM-MSCs | NA | NA | Spinner flasks (40, 45, 50 rpm)/4 × 10^5^ cells/mL | 5 days  (24 h) | αMEM, 10% FBS, 100 units/mL penicillin, 100 μg/mL streptomycin |
| Miranda JP *et al*. *Front Immunol* 2019;10:18  [9] | Dynamic | Human  UC-MSCs | NA | NA | Spinner flasks (80 rpm for formation, 110 rpm for maintenance)/1 × 10^6^ cells/mL | 7 days  (2 days) | αMEM, 15% FBS |
| Allen LM *et al*. *Stem Cells Int* 2019;2019:4607461  [26] | Dynamic | Human  Syf-MSCs | NA | NA | Spinner flasks (70 rpm)/5 × 10^4^ cells/mL | 12 days  (6 days) | Serum-free medium |
| Niibe K *et al*. *Front Bioeng Biotechnol*  2020;8:590332  [20] | Dynamic | Mouse/Human  BM-MSCs | NA | NA | Shaker flasks (85-95 rpm)/5 × 10^5^ cells/mL | 2 months  (9 days) | Mouse MSCs: GlutaMAX αMEM, 10% FBS, 1% P/S, 10 mM HEPES  Human MSCs: DMEM, 10% FBS, 1% P/S, 10 mM HEPES, 20 ng/mL FGF-2 |
| Ohori–Morita Y *et al*. *Stem Cells Transl Med* 2022;11:434–449  [21] | Dynamic | Human  BM-MSCs | NA | NA | Shaker flasks (85-95 rpm)/5 × 10^5^ cells/mL | 1 month  (3 days) | Advanced DMEM, 1% P/S, 1% L-glutamine, 10 mM HEPES, 20 ng/mL recombinant human epidermal growth factor, 20 ng/mL recombinant human basic fibroblast growth factor, 2% N-2, 2% B27 |
| Shimazawa Y *et al*. *Biotechnol J* 2022;17:e2100137  [28] | Dynamic | Mouse  AD-MSCs | NA | NA | Agarose microwell plate in 6 well plate on rotation platform (60 rpm)/2 × 10^6^ cells/well | 3 days  (24 h) | DMEM, 20% FBS, P/S-glutamine mixed solution |
| Park JH *et al*. *Biomater Res*  2023;27:e2100137  [29] | Dynamic | Human  MSCs (no cell type mentioned) | NA | NA | Anti-gravity bioreactor (resonance frequency of 1.6 MHz)/1 × 10^6^ cells/device | 24 h  (12 h) | DMEM, 10% FBS, 1% P/S |

**Abbreviations:** AD-MSCs (adipose tissue-derived mesenchymal stromal/stem cells), BM-MSCs (bone marrow-derived mesenchymal stromal/stem cells), Syf-MSCs (synovial fluid-derived mesenchymal stromal/stem cells), UC-MSCs (umbilical cord-derived mesenchymal stromal/stem cells), DMEM-LG (DMEM-low glucose), P/S (penicillin-streptomycin), NA (not applicable)
