# Supplementary material for: Current perspectives on the dynamic culture of mesenchymal stromal/stem cell spheroids
Source: Stem Cells Transl Med. 2024 Dec 31;14(3):szae093. doi: 10.1093/stcltm/szae093 (PMC11954588; doi:10.1093/stcltm/szae093)
Supplement: szae093_suppl_Supplementary_Table_S3 [file szae093_suppl_supplementary_table_s3.docx]

**Supplementary Table 3. Animal studies and clinical trials using MSC spheroids**

| References  [reference number] | Method | Cell type | Static culture/initial cell number | Static culture duration | Dynamic culture （agitation speed）/initial cell number | Dynamic culture duration | Medium | Animal model | Defect/disease model | Method of administration/transplant form/application volume | Outcomes |
| --- | --- | --- | --- | --- | --- | --- | --- | --- | --- | --- | --- |
| Animal studies | | | | | | | | | | | |
| Bhang SH *et al*. *Biomaterials* 2011;32:2734–2747  [13] | Dynamic | Human  AD-MSCs | NA | NA | Spinner flasks (70 rpm)/6 × 10^5^ cells/mL | 3 days | GM: αMEM, 10% FBS, 100 units/mL penicillin, 100 μg/mL streptomycin | Mouse | Induced hindlimb ischemia model | Intramuscular injection/Spheroids/1 × 10^7^ cells | MSC spheroids showed improved cell survival, angiogenic factor secretion, neovascularization, and limb survival as compared to monolayer MSCs grafted as dissociated cells. |
| Zhang Q *et al*. *Stem Cells Dev* 2012;21:937–947 | Static | Human  Gingival-MSCs | Ultra-low attachment dish/2 × 10^5^ cells/mL | 3 days | NA | NA | GM: αMEM, 10% FBS, 100 units/mL penicillin, 100 µg/mL streptomycin, 2 mM L-glutamine, 100 mM nonessential amino acid, 550 µM 2-mercaptoethanol | Mouse | Induced mucositis model/tongue samples | Intravenous injection/Spheroid derived cell suspension/1 × 10^6^ cells | Treatment with MSC spheroids led to faster regeneration of the basal layer and greater restoration of the epithelial layer compared to the treatment with monolayer MSCs. |
| Santos JM *et al*. *Stem Cell Res Ther* 2015;6:90  [8] | Dynamic | Human  UC-MSCs | NA | NA | Spinner flasks (80 rpm for formation, 110 rpm for maintenance)/1 × 10^6^ cells/mL | 2 days | CM: αMEM, 2mM L-glutamine, 1 g/L glucose, 2.2 g/L sodium bicarbonate, 10% FBS | Rat | Full-thickness cutaneous wounds (each animal 4 wounds) | Subcutaneous injection/CM/100 μL administration repeated in a total of 300 μl | Complete wound closure in wounds treated with CM was significantly faster than the control. |
| Suenaga H *et al*. *J Mater Sci Mater Med* 2015;26:254  [60] | Dynamic | Human  BM-MSCs | NA | NA | Rotary orbital shaker (70 rpm)/1 × 10^7^ cells/mL | 1 day | OS: DMEM, 10% FBS, 100 nM dexamethasone, 0.05 mM l-ascorbic acid-2-phosphate, 10 mM sodium glycerophosphate, 100 units/mL penicillin, 100 μg/mL streptomycin, 0.25 μg/mL amphotericin B | Rat | Calvarial defect (8 mm) | Direct application/Spheroids/12.5 mm^3^ | MSC spheroids group showed the best bone formation in both µ-CT and histological H&E analysis. Highest bone strength and elasticity of new bones in the MSC spheroids group were 50 and 60 % of the natural bone, respectively, with similar chemical composition to the natural bone. |
| Xu Y *et al*. *J* *Cell Mol Med* 2016;20:1203–1213  [31] | Static | Human  AD-MSCs | Hanging drops/35 μL drops of medium containing 2.5 × 10^4^ cells | 3 days | NA | NA | GM: αMEM, 10% FBS | Rat | Ischemic kidney model | Injection to the kidney cortex/Spheroid derived cell suspension/2 × 10^6^ cells | Compared to monolayer cultured cells, spheroids-derived cells were more beneficial in protecting the damaged kidney against apoptosis, reducing tissue damage, promoting vascularization and improving renal function. |
| Miranda JP *et al*. *Front Immunol* 2019;10:18  [9] | Dynamic | Human  UC- MSCs | NA | NA | Spinner flasks (80 rpm for formation, 110 rpm for maintenance)/1 × 10^6^ cells/mL | 2 days | CM: αMEM | Rat | Adjuvant-induced model for arthritis (AIA) | Intraarticular injection/CM/100 μL administration repeated in a total of 400 μL | Significantly higher therapeutic potential was observed by spheroids-derived CM than monolayer-derived CM. |
| Kouroupis D *et al*. *Stem Cell Res Ther* 2021;12:44 | Static | Human  IFP-MSCs | Gas-permeable 6 well plates/2 × 10^5^ cells/well | 2 days | NA | NA | GM: DMEM, 10% FBS, methylcellulose solution (4/1 ratio) | Rat | Induced acute synovial/IFP inflammation knee model | Single intraarticular knee joint injection/Spheroids/5 × 10^5^ cells | MSC spheroids injection significantly reduced the synovitis and IFP fibrosis that was further reversed up to day 28. |
| Li Y *et al*. *Stem Cell Res Ther* 2021;12:358  [56] | Dynamic | Human  AM-MSCs | NA | NA | Spinner flasks/NA | 60 h | GM: MEM, 5% EliteGro™-Adv | Rat | Middle cerebral artery occlusion (MCAO) and reperfusion | Intravenous injection/Spheroid derived cell suspension/1–3 × 10^6^ cells | Spheroids-derived cells significantly reduced the infarct volume of the brain with increased engraftment of the cells into the ischemic tissue, compared to monolayer-cultured cells. |
| Lee N *et al*. *Fron Immunol*  2022;13:940258 | Static | Human  Tonsillar- MSCs | Hanging drops/1 × 10^4^ cells/spheroid | 3 days | NA | NA | GM: αMEM, 10% FBS, 1% P/S | Mouse | Induced neuropathic pain murine model | Intramuscular injection (gastrocnemius muscle) nearby injured nerve/Spheroids/100 spheroids | Transplantation of MSC spheroids was more effective than monolayer MSCs in alleviating chronic neuropathic pain and reducing the expression of proinflammatory cytokines. |
| Ohori–Morita Y *et al*. *Stem Cells Transl Med* 2022;11:434–449  [21] | Dynamic | Human  BM-MSC | NA | NA | Shaker flasks (85–95 rpm)/5 × 10^5^ cells/mL | 21 days | NM: advanced DMEM, 1% P/S, 1% L-glutamine, 10 mM HEPES, 20 ng/mL recombinant human epidermal growth factor, 20 ng/mL recombinant human basic fibroblast growth factor, 2% N-2, 2% B27 | Rat | Femur rectangular defect (3 mm × 5 mm) | Direct application/Spheroids/1 × 10^6^ cells | Thick and contiguous mineralized structure was observed in the defects implanted with NM cultured spheroids, where the defect was nearly completely closed with higher average bone mineral density and bone volume in comparison with the control. |
| Shimazawa Y *et al*. *Biotechnol J* 2022;17:e2100137  [28] | Dynamic | Mouse  AD-MSCs | NA | NA | Agarose microwell plate in 6 well plate on rotation platform (60 rpm)/2 × 10^6^ cells/well | 1 day | GM: DMEM, 20% FBS, P/S-glutamine mixed solution | Mouse | LPS-induced inflammatory model | Intravenous injection/Spheroids/850 spheroids | Intravenous injection of MSC spheroids improved the delivery efficiency of the cells to the lung and prolonged their survival after intravenous injection and decreased the interleukin-6 and tumor necrosis factor-α. |
| Wu YC *et al*. *Sci Rep* 2022;12:1227  [57] | Dynamic | Human  AD-MSCs | NA | NA | Spinner flasks (40 rpm)/1.5 × 10^5^ cells/mL | NM | GM: DMEM, 10% FBS and 1% P/S | Rat | Induced liver cirrhosis model | Direct application/Spheroids/3 spheroids | Hybrid MSCs/hepatocyte spheroids consistently exhibited a beneficial effect in ameliorating fibrotic and potentially preserving liver function. |
| Esmaeili A *et al*. *Sci Rep* 2022;12:19827  [58] | Dynamic | Rabbit  BM-MSCs | NA | NA | Spinner flasks (40 rpm for formation of the chondrocytes aggregates, 45 rpm for formation of MSC aggregates and MSC-chondrocytes co-aggregates, 60 rpm for maintenance)/5 × 10^5^ cells/mL | 7 days | GM: DMEM, 10% FBS | Rat | Osteoarthritis model (knee) | Intraarticular injection/Extracellular vesicles (EV)/50 μg EV | MSC-chondrocytes co-aggregation could elevate EV therapeutic properties and provide a new potential for the treatment of diseases including osteoarthritis. |
| Gangadaran P *et al*. *Biochem Biophys Res Commun* 2023;673:87–95 | Static | Human  BM-MSC | 96 well non adhesive plates/1 × 10^4^ cells/well | 5 days | NA | NA | CM: DMEM-F12 | Mouse | 1.5 cm full thickness burn wound | Direct application/Spheroid derived cell suspension/1 × 10^6^ cells | MSC spheroids-treated skin sections showed smaller wounds, more regenerated hair follicles, increased collagen deposition and maturation compared with monolayer MSCs- and control-treated wounds. |
| Hisamatsu D *et al*. *Pharmaceutics* 2023;15 | Static | Mouse  AD-MSCs | EZSPHERE microwell plates (AGC Techno Glass)/NA | 2 days | NA | NA | GM: GlutaMAX DMEM, 20% FBS, 20 ng/mL basic fibroblast growth factor, 1% P/S | Mouse | Induced colitis model | Trans anal administration/Spheroids/1 × 10^6^ cells | CD73^+^ cell spheroids showed enhanced engraftment at the injured site through the enteral route, facilitated extracellular matrix remodeling, and downregulated inflammatory gene expression in fibroblasts, leading to the attenuation of colonic atrophy. |
| Tu CC *et al*. *J Periodontol* 2024;95:372–383 | Static | Rat  Alveolar mucosal- MSCs | Agarose microwell plate in 12 well plate/2 × 10^5^ cells/well | 3 days | NA | NA | GM: αMEM, 10% FBS, 100 units/mL P/S  or OS: GM with 10 nM dexamethasone, 50 μM ascorbic acid 2-phosphate, 10 μM β-glycerophosphate | Rat | Extraction socket of bilateral maxillary first molars | Direct application/Spheroids/1.2 × 10^6^ cells | OS spheroids-treated group showed less wound dehiscence, faster full epithelization, and better healing index. Collagen matrix deposition was more prominent on day 7 and corticalized bone was filled in the socket on day 28 in the OA spheroids-treated group. |
| Clinical trials | | | | | | | | | | | |
| Lee DH *et al*. *Int J Mol Sci*  2023;24:16827 | Static | Human  AD-MSCs | EZSPHERE microwell plates (AGC Techno Glass)/3 × 10^4^ cells/cm^2^ | 1 day | NA | NA | DMEM, 10% FBS, 10 ng/mL recombinant matrilin-3 | NA | Intervertebral disc (IVD) degeneration; Single arm clinical trial (8 patients) | Direct application/Spheroids/6 × 10^6^ cells | 6 out of 8 patients showed improvements in pain score and disability score. Some improvements in MRI findings were also observed in a subset of patients. |
| Özkan B *et al*. *Stem Cell Res Ther* 2023;14:252 | Static | Human  UC- MSCs | 96 well non adhesive plates/1 × 10^5^ cells/well | 48 h | NA | NA | Serum-free medium (MSC nutristem® XF Medium), 1% ciprofloxacin | NA | Retinitis pigmentosa (RP); Non randomized clinical trial (15 patients) | Direct application/Spheroids/45 spheroids | Significant improvement was observed in visual acuity (80% of the patients) and visual fields of the patients. |

**Abbreviations:** AD-MSCs (adipose tissue-derived mesenchymal stromal/stem cells), AM-MSCs (amnion membrane-derived mesenchymal stromal/stem cells), BM-MSCs (bone marrow-derived mesenchymal stromal/stem cells), IFP-MSCs (infrapatellar fat pad-derived mesenchymal stromal/stem cells), UC-MSCs (umbilical cord mesenchymal stromal/stem cells), GM (growth medium), OS (osteogenic medium), CM (conditioned medium), NM (neural crest medium), NA (not applicable)
